# Diabetes Mellitus Is Associated With Increased Risk for Subsequent Hip Arthroscopy After Index Hip Arthroscopy

**DOI:** 10.1002/ars2.70105

**Published:** 2026-10-16

**Authors:** Nicholas J. Morriss, Justin Harrington, Jacob Waldron, Brian Giordano

**Affiliations:** ^1^ Department of Orthopaedics University of Rochester Medical Center Rochester New York U.S.A.

## Abstract

**Purpose:**

To determine whether diabetes mellitus is associated with higher systemic and hip‐specific complications and to evaluate reoperation rates after hip arthroscopy.

**Methods:**

Using the TriNetX Research Network and Current Procedural Terminology and International Classification of Diseases codes, we identified diabetic and nondiabetic patients aged 12 to 65 years who underwent hip arthroscopy between 2005 and 2025. Ninety‐day systemic complications and 6‐month, 1‐year, and 3‐year hip‐specific outcomes were evaluated. Propensity score matching (1:1) was performed on 35 demographic and comorbidity variables. Risk analyses, Kaplan‐Meier curves, and Cox proportional hazards models compared outcomes, with significance set at *α* = 0.05.

**Results:**

After matching, 1784 diabetic and 1784 nondiabetic patients were included. At 90 days, diabetes was associated with a higher hospital admission rate (odds ratio [OR] 1.83, 95% confidence interval [CI] 1.16‐2.87, *P* = .01). Diabetic patients had higher rates of subsequent hip arthroscopy at 1 year (OR 1.42, 95% CI 1.12‐1.79, *P* = .004) and 3 years (OR 1.39, 95% CI 1.14‐1.69, *P* = .001). Rates of arthroscopic irrigation and debridement were higher in patients with diabetes at 6 months (OR 1.81, 95% CI 1.09‐3.00, *P* = .019), 1 year (OR 1.96, 95% CI 1.32‐2.92, *P* = .001), and 3 years (OR 1.75, 95% CI 1.29‐2.37, *P* < .001). Infection diagnoses were also higher among diabetic patients at 6 months (OR 1.64, 95% CI 1.06‐2.52, *P* = .033) and 3 years (OR 1.70, 95% CI 1.24‐2.33, *P* = .008).

**Conclusions:**

Diabetes was not associated with higher rates of most early systemic complications after hip arthroscopy, although hospital admissions were more common among patients with diabetes. Diabetes was associated with higher rates of subsequent hip arthroscopy procedures, arthroscopic irrigation and debridement, and infection diagnoses after index hip arthroscopy; however, these findings should be interpreted in the context of database limitations, including the inability to confirm laterality or ipsilateral revision status.

**Level of Evidence:**

Level III, retrospective cohort study.

The use of hip arthroscopy has grown rapidly over the past decade, with an 85% increase in procedural volume in the United States and a steady shift toward outpatient surgery.^1^ Although major complications remain rare, as overall case numbers continue to rise, even small increases in adverse events become meaningful, creating a need to identify patient characteristics that increase the risk of perioperative complications to allow more informed discussion between patient and surgeon.

Diabetes mellitus has been identified as a comorbidity that may increase perioperative risk in patients undergoing orthopaedic surgery. Diabetes is common among patients undergoing orthopaedic procedures and is associated with higher rates of infection, impaired wound healing, and medical complications throughout arthroscopic and other orthopaedic surgeries.^2^, ^3^ As the incidence of diabetes in patients undergoing orthopaedic surgery continues to rise,^4^ it becomes increasingly important to identify the impact of diabetes on outcomes after orthopaedic surgery.^2^, ^5^, ^6^ Evidence specific to hip arthroscopy, however, remains limited and mixed. A matched cohort study found that patients with diabetes experienced similar improvements in patient‐reported outcomes at 2 years postoperatively but observed increased complication and revision rates compared with those of nondiabetic controls that did not reach statistical significance.^7^ Although broader sports medicine literature suggests that diabetes increases short‐term morbidity and readmissions after arthroscopic procedures, with a mixed influence on long‐term outcomes, there is a dearth of literature evaluating the impact of diabetes on outcomes after hip arthroscopy.^2^, ^8^ The currently published data do not clearly establish whether diabetes increases complications after hip arthroscopy.

This investigation used a large, federated electronic health‐record network to evaluate the population‐level impact of diabetes mellitus on outcomes after hip arthroscopy. The purposes of this study were to determine whether diabetes mellitus is associated with higher systemic and hip‐specific complication and to evaluate reoperation rates after hip arthroscopy. Our hypotheses were that diabetes would be associated with higher short‐term complication rates, an increased long‐term likelihood of subsequent hip arthroscopy and higher rates of subsequent arthroplasty.

## METHODS

A retrospective deidentified database cohort study was performed to evaluate the association between pre‐existing diabetes mellitus and adverse outcomes after hip arthroscopy. Data were obtained from the TriNetX Research Network, a federated electronic health record network composed of participating health care organizations that contribute deidentified patient‐level demographic, diagnostic, procedural, laboratory, and encounter data. Patients in this analysis therefore represent individuals receiving care within participating health systems rather than a single‐institution surgical registry. Ethical approval for this study was waived by the University of Rochester Institutional Review Board as this was deemed a deidentified study.

Patients who underwent hip arthroscopy between October 1, 2005, and November 19, 2025, were identified using the Current Procedural Terminology hip arthroscopy family of codes, including 29860, 29861, 29862, 29863, 29914, 29915, and 29916. Eligible patients were 12 to 65 years old at the time of the index procedure. Two cohorts were constructed: a diabetes cohort, defined by a diagnosis code for diabetes mellitus (ICD‐10 E08‐E13) at any time, and a control cohort with no diabetes codes. The index event was the first qualifying hip arthroscopy, and patients whose index procedure occurred more than 20 years before the analysis date were excluded per platform rules.

Outcomes were defined a priori based on clinical relevance and were organized, where possible, using the Clavien‐Dindo‐Sink framework for surgical complication severity. Because TriNetX captures diagnosis and procedure codes rather than granular clinical treatment details, formal grade assignment was limited to outcomes that could be reasonably classified by the type of event or intervention. General systemic complications were evaluated within 90 days after surgery and included emergency department visits, hospital admissions, thromboembolic events, cardiopulmonary complications, renal failure, transfusion, wound disruption, and mortality, using the definitions listed in Table 1. Hip‐specific outcomes, defined in Table 2, were separately evaluated and included subsequent hip‐related outcomes (chondroplasty or labral resection, synovectomy, femoroplasty, acetabuloplasty, and labral repair), arthroscopic irrigation and debridement (subsequent arthroscopy with debridement or synovectomy, open incision and drainage, and soft tissue or osseous debridement around the hip), infection outcomes (postoperative infection, septic arthritis, osteomyelitis, sepsis, infectious bursitis, and pathogen‐specific hip infections), subsequent labral repair, and subsequent acetabular or femoral work. Subsequent hip‐related procedural and infection outcomes were assessed after the index procedure at 6 months and 1 and 3 years. Because procedure laterality is not available within TriNetX, these outcomes should be interpreted as subsequent hip‐related diagnoses or procedures after index hip arthroscopy, rather than confirmed ipsilateral revision procedures. Similarly, infection diagnoses occurring beyond the early postoperative period should be interpreted as subsequent coded infection diagnoses after index hip arthroscopy rather than confirmed surgical‐site infections attributable to the index procedure.

**TABLE 1:** Outcome Definitions for Systemic Postoperative Outcomes

**Outcome**	**Approximate Clavien‐Dindo‐Sink Category**	**Code**	**Definition**
ED visit	Grade I/II, depending on evaluation and treatment required	99281	ED service, Level I (minimal complexity)
		99282	ED service, Level II (low complexity)
		99283	ED service, Level III (moderate complexity)
		99284	ED service, Level IV (moderate‐high complexity)
		99285	ED service, Level V (high complexity)
Hospital visit	Grade II/III, depending on admission indication and intervention	99221	Initial hospital care, Level I (low complexity)
		99222	Initial hospital care, Level II (moderate complexity)
		99223	Initial hospital care, Level III (high complexity)
Mortality	Grade V	R99.xx	Ill‐defined and unknown causes of mortality
Wound disruption	Grade I‐III, depending on treatment required	T81.3	Disruption or dehiscence of surgical wound
MI	Grade II‐IV	I21	Acute MI
		I22	Subsequent ST elevation MI and non‐ST elevation MI
DVT	Grade II	I82.4	Acute embolism/thrombosis of deep veins of lower extremity
		I82.5	Chronic embolism/thrombosis of deep veins of lower extremity
Pneumonia	Grade II‐IV	J13	Pneumonia because of *Streptococcus*
		J14	Pneumonia because of *H. influenzae*
		J15	Bacterial pneumonia
		J16	Pneumonia because of other infectious organisms
		J17	Pneumonia in diseases classified elsewhere
		J18	Pneumonia, unspecified
		483	ICD‐9: pneumonia, organism unspecified
Acute renal failure	Grade II‐IV	N17	Acute kidney failure, all subtypes
Acute posthemorrhagic anemia	Grade II if transfusion or pharmacologic treatment required	D62	Acute blood loss anemia
CVA	Grade IV	I61	Intracerebral hemorrhage
		I62	Nontraumatic intracranial hemorrhage
		I63	Cerebral infarction
		I66	Occlusion or stenosis of cerebral arteries
Transfusion	Grade II	36430	Blood transfusion service
PE	Grade II‐IV	I26	PE
Respiratory failure	Grade IV	J95	Respiratory complications after medical care or surgery
		J96	Acute and chronic respiratory failure
		J98.19	Other pulmonary collapse
Atelectasis	Grade I/II	J98.11	Atelectasis
UTI	Grade II if treated with antibiotics	N39.0	Urinary tract infection, site not specified
Hardware removal	Grade III	20670	Removal of superficial implant
		20680	Removal of deep implant

*Note*: This table lists systemic postoperative outcomes evaluated at 90 days postoperatively and the corresponding ICD‐10 and CPT codes used to identify each event within the database. Outcomes are grouped by clinical category, and each code is presented in an individual subrow with its full diagnostic or procedural definition. ED visits and hospital visits were identified using evaluation and management service codes. Medical and surgical complications, including infection, wound disruption, thromboembolic events, respiratory complications, renal failure, anemia, CVAs, and UTIs, were captured using ICD‐10 diagnosis codes. Procedural outcomes, including transfusion and hardware removal, were captured using CPT codes.

CPT, Current Procedural Terminology; CVA, cerebrovascular accident; DVT, deep vein thrombosis; ED, emergency department; *H. influenza*, *Haemophilus influenza*; ICD, International Classification of Diseases; MI, myocardial infarction; PE, pulmonary embolism; UTI, urinary tract infection.

**TABLE 2:** Hip‐Related Outcome Definitions

**Outcome**	**Code**	**Description**
Subsequent arthroscopy	CPT 29862	Arthroscopy, hip; debridement/shaving of articular cartilage, abrasion arthroplasty, and/or labral resection
	CPT 29863	Arthroscopy, hip; synovectomy
	CPT 29914	Arthroscopy, hip; femoroplasty (cam lesion)
	CPT 29915	Arthroscopy, hip; acetabuloplasty (pincer lesion)
	CPT 29916	Arthroscopy, hip; labral repair
Irrigation and debridement	CPT 29862	Arthroscopy, hip; debridement/shaving/labral resection
	CPT 29863	Arthroscopy, hip; synovectomy
	CPT 1004843	Incision and drainage, pelvis/hip joint
	CPT 11042	Debridement, subcutaneous tissue; first 20 cm^2^
	CPT 11043	Debridement, muscle/fascia; first 20 cm^2^
	CPT 11044	Debridement, bone; first 20 cm^2^
	CPT 27030	Arthrotomy, hip, with drainage (infection)
	CPT 27301	I&D, deep abscess/bursa/hematoma, thigh or knee
Diagnosis of infection	ICD‐10 T81.4	Infection after a procedure
	ICD‐10 T84.5	Infection because of internal joint prosthesis
	ICD‐10 M00	Pyogenic arthritis
	ICD‐10 M86	Osteomyelitis
	ICD‐10 A41	Other sepsis
	ICD‐10 M01.X5	Direct infection of hip in infectious/parasitic disease
	ICD‐10 M01.X59	Direct infection of unspecified hip
	ICD‐10 M71.15	Other infective bursitis, hip
	ICD‐10 M00.05	Staphylococcal arthritis, hip
	ICD‐10 M00.15	Pneumococcal arthritis, hip
	ICD‐10 M00.25	Other streptococcal arthritis, hip
	ICD‐10 M00.85	Arthritis because of other bacteria, hip
Subsequent labral repair only	CPT 29916	Arthroscopy, hip; labral repair
Subsequent bony work	CPT 29914	Arthroscopy, hip; femoroplasty
	CPT 29915	Arthroscopy, hip; acetabuloplasty

*Note*: This table lists hip‐related postoperative outcomes evaluated at 6 months, 1 year, and 3 years, and the corresponding ICD‐10 and CPT codes used to identify each event within the database. Outcomes are grouped by clinical category, and each code is presented in an individual subrow with its full diagnostic or procedural definition. Procedural outcomes, including subsequent arthroscopy, irrigation and debridement, labral repair, and bony work, were captured using CPT codes, whereas infection was captured using ICD codes.

CPT, Current Procedural Terminology; I&D, irrigation and debridement; ICD, International Classification of Diseases.

Hemoglobin A1c values recorded within 3 months of the index arthroscopy were identified for descriptive analysis. A hemoglobin A1c below 7.0% was considered generally consistent with adequate glycemic control based on contemporary diabetes management guidelines.^9^ Because the diabetes cohort differed from controls in demographic and comorbidity profiles, the 1:1 nearest‐neighbor propensity score matching without replacement that is built into the TriNetX analytics environment (caliper 0.1 standard deviation of the logit) was performed. The model included age, sex, race, ethnicity, and baseline comorbidities (malignancy, anemia, coagulation disorders, hypothyroidism, obesity, electrolyte and acid‐base disorders, chronic liver disease, chronic obstructive pulmonary disease, chronic kidney disease, hypertension and cardiovascular disease, asthma, chronic pain, mood and anxiety disorders, substance use disorders, nicotine dependence, and congenital hip deformities). TriNetX generates postmatching cohort characteristics and balance diagnostics for each matched variable. Covariate balance after matching was assessed using standardized mean differences, with values less than 0.10 considered acceptable.

### Statistical Analysis

An a priori power analysis was performed to determine the sample size required to detect a difference in proportions between the 2 cohorts, based on results by Quesada‐Jimenez et al. evaluating revision arthroscopy in hip arthroscopy between diabetic and nondiabetic patients.^10^ In the diabetes cohort, 24.1% of diabetic hips required revision arthroscopy, compared with 9.2% in controls. With a 2‐sided *α* of 0.05, and a desired power of 80%, the calculated effect size corresponded to a 14.9% absolute difference between groups. Based on these inputs, the required sample size was approximately 194 patients per cohort to achieve adequate power for detecting this difference in outcomes. Risk analyses were used to calculate event proportions with risk differences, risk ratios, and odds ratios (ORs) with 95% confidence intervals (CIs). To preserve patient confidentiality, TriNetX inherently censors outcome results where 10 or fewer patients in both cohorts experienced that outcome. Therefore, statistical analysis was not performed on results with 10 or fewer patients per cohort. Time‐to‐event differences were evaluated using Kaplan‐Meier survival curves with log‐rank tests and hazard ratios estimated by Cox proportional hazards models. Number of instances analyses were used descriptively to summarize the distribution of subsequent procedures and infections. Statistical significance was defined as *P* < .05.

## RESULTS

### Propensity Matching and Glycemic Control

Before matching, the diabetes cohort included 1846 patients and the control cohort included 33,780 patients. After 1:1 nearest‐neighbor matching, 1784 patients remained in each cohort. The results of propensity score matching are presented in Table S1. All standardized mean differences for matched variables were less than 0.10, showing adequate covariate balance between groups. Within the diabetic cohort, 872 (48%) had a recorded A1c within 3 months of arthroscopy, and the mean hemoglobin A1c was 6.59 ± 1.64.

### Systemic Complications at 90 Days

At 90 days after index hip arthroscopy, hospital admissions were more common in the diabetes cohort (3.0% vs 1.7%; OR 1.83, 95% CI 1.16‐2.87, *P* = .01). Emergency department visits were not statistically different (6.2% of vs 5.3%, OR 1.18, 95% CI 0.89‐1.56, *P* = .34) (Table 3). Deep vein thrombosis occurred at comparable frequencies (0.8% vs 0.7%; OR 1.17, 95% CI 0.54‐2.53, *P* = .74), as did acute posthemorrhagic anemia (1.2% vs 0.8%; OR 1.51, 95% CI 0.76‐2.97, *P* = .25). For all other systemic complications, including mortality, wound disruption, myocardial infarction, pneumonia, acute renal failure, cerebrovascular accident, transfusion, pulmonary embolism, respiratory failure, atelectasis, urinary tract infection, and hardware removal, TriNetX censored results because fewer than 10 events occurred in each group, and statistical analyses were not performed.

**TABLE 3:** Ninety‐Day Systemic Outcomes After Hip Arthroscopy as a Function of Diabetes

**Outcome**	**Diabetes N (%) (N = 1791)**	**Control N (%) (N = 1791)**	**Risk Ratio (95% CI)**	**Odds Ratio (95% CI)**	**Log‐Rank** ***P***	**Hazard Ratio (95% CI)**	***P*** **(HR)**
ED visit	111 (6.20%)	95 (5.30%)	1.17 (0.90‐1.52)	1.18 (0.89‐1.56)	.34	1.14 (0.87‐1.50)	.83
Hospital visit	54 (3.02%)	30 (1.67%)	1.80 (1.16‐2.80)	1.83 (1.16‐2.87)	.01	1.78 (1.14‐2.77)	.72
DVT	14 (0.78%)	12 (0.67%)	1.17 (0.54‐2.52)	1.17 (0.54‐2.53)	.74	1.14 (0.53‐2.47)	.34
Acute posthemorrhagic anemia	21 (1.17%)	14 (0.78%)	1.50 (0.77‐2.94)	1.51 (0.76‐2.97)	.25	1.48 (0.75‐2.92)	.63

*Note*: Ninety‐day general systemic complications in the diabetes and matched control cohorts. Values are presented as counts with percentages, along with odds ratios and corresponding 95% CIs. *P* values reflect the comparison of odds between groups.

CI, confidence interval; DVT, deep vein thrombosis; ED, emergency department; HR, haxa.

### Hip‐Related Outcomes

At 6 months, rates of subsequent coded hip arthroscopy were similar between groups (6.2% vs 4.8%; OR 1.30, 95% CI 0.97‐1.73, *P* = .078) but were higher in the diabetes cohort at 1 year (10.1% vs 7.3%; OR 1.42, 95% CI 1.12‐1.79, *P* = .004) and 3 years (14.9% vs 11.2%; OR 1.39, 95% CI 1.14‐1.69, *P* = .001) (Table 4). Arthroscopic irrigation and debridement procedures were more common in the diabetes cohort at 6 months (2.4% vs 1.3%; OR 1.81, 95% CI 1.09‐3.00, *P* = .019), 1 year (4.1% vs 2.1%; OR 1.96, 95% CI 1.32‐2.92, *P* = .001), and 3 years (6.7% vs 3.9%; OR 1.75, 95% CI 1.29‐2.37, *P* < .001). Coded infection diagnoses, defined using the International Classification of Diseases codes listed in Table 2, were similar at 1 year (3.8% vs 2.8%; OR 1.38, 95% CI 0.95‐2.01, *P* = .144) but were more common in the diabetes cohort at 6 months (3.1% vs 1.9%; OR 1.64, 95% CI 1.06‐2.52, *P* = .033) and 3 years (5.9% vs 3.6%; OR 1.70, 95% CI 1.24‐2.33, *P* = .008). Rates of subsequent labral repair procedures were similar at 6 months (4.1% vs 3.9%; OR 1.04, 95% CI 0.75‐1.46, *P* = .798), 1 year (6.2% vs 5.8%; OR 1.08, 95% CI 0.82‐1.43, *P* = .573), and 3 years (9.0% vs 8.1%; OR 1.13, 95% CI 0.89‐1.43, *P* = .309). Subsequent coded acetabular or femoral procedures were similar at 6 months (4.8% vs 4.1%; OR 1.19, 95% CI 0.86‐1.63, *P* = .292) and 1 year (7.5% vs 6.0%; OR 1.27, 95% CI 0.98‐1.66, *P* = .072) but were more common in the diabetes cohort at 3 years (11.3% vs 8.8%; OR 1.32, 95% CI 1.06‐1.65, *P* = .012).

**TABLE 4:** Hip‐Related Outcomes After Hip Arthroscopy as a Function of Diabetes

**Outcome**	**Group**	**6 mo**		**1 yr**		**3 yr**	
		**Diabetes**	**Control**	**Diabetes**	**Control**	**Diabetes**	**Control**
Subsequent arthroscopy	N	110	86	180	131	266	200
	Risk, %	6.20	4.80	10.10	7.30	14.90	11.20
	Risk ratio	1.28 (0.97‐1.68)		1.37 (1.11‐1.70)		1.33 (1.12‐1.58)	
	Odds ratio	1.30 (0.97‐1.73)		1.42 (1.12‐1.79)		1.39 (1.14‐1.69)	
	*P* value	.078		**.004**		**.001**	
Irrigation and debridement	N	110	86	180	131	266	200
	Risk, %	2.40	1.30	4.10	2.10	6.70	3.90
	Risk ratio	1.79 (1.09‐2.94)		1.92 (1.30‐2.83)		1.70 (1.27‐2.27)	
	Odds ratio	1.81 (1.09‐3.00)		1.96 (1.32‐2.92)		1.75 (1.29‐2.37)	
	*P* value	**.019**		**.001**		**<.001**	
Diagnoses of infection	N	55	34	67	49	106	64
	Risk, %	3.08	1.91	3.76	2.75	5.94	3.59
	Risk ratio	1.62 (1.06‐2.47)		1.37 (0.95‐1.96)		1.66 (1.22‐2.24)	
	Odds ratio	1.64 (1.06‐2.52)		1.38 (0.95‐2.01)		1.70 (1.24‐2.33)	
	*P* value	**.033**		.144		**.008**	
Subsequent labral repair	N	73	70	111	103	161	144
	Risk, %	4.10	3.90	6.20	5.80	9.00	8.10
	Risk ratio	1.04 (0.76‐1.44)		1.08 (0.83‐1.40)		1.12 (0.90‐1.39)	
	Odds ratio	1.04 (0.75‐1.46)		1.08 (0.82‐1.43)		1.13 (0.89‐1.43)	
	*P* value	.798		.573		.309	
Subsequent acetabuloplasty or femoroplasty	N	86	73	134	107	202	157
	Risk, %	4.80	4.10	7.50	6.00	11.30	8.80
	Risk ratio	1.18 (0.87‐1.60)		1.25 (0.98‐1.60)		1.29 (1.06‐1.57)	
	Odds ratio	1.19 (0.86‐1.63)		1.27 (0.98‐1.66)		1.32 (1.06‐1.65)	
	*P* value	.292		.072		**.012**	

*Note*: This table reports outcomes of hip arthroscopy at 6‐month, 1‐year, and 3‐year follow‐up, comparing patients with diabetes to matched controls. Risk, risk ratio, and odds ratio values compare the incidence of each outcome between cohorts at each interval. Bolded values indicate statistical significance at *P* < .05.

mo, month; yr, year.

## DISCUSSION

The most important finding of this study was that no significant differences were observed for most analyzable 90‐day systemic outcomes between patients with and without diabetes, and overall rates of serious medical events were low. Hospital admission was the only systemic outcome that differed significantly between groups. Emergency department visits, deep vein thrombosis, and acute posthemorrhagic anemia did not significantly differ between cohorts. Diabetes was also associated with higher rates of subsequent hip arthroscopy procedures, arthroscopic irrigation and debridement, and infection diagnoses after index hip arthroscopy, but these hip‐related outcomes cannot be confirmed as ipsilateral complications or revisions because laterality is unavailable in TriNetX. Prior work examining diabetes in this setting has been limited to small, single‐center series with modest diabetic sample sizes and a primary focus on patient‐reported outcomes rather than complications or reintervention burden.^7^, ^10^ The present study was able to evaluate outcomes and risk of subsequent arthroscopy as well as infection burden after arthroscopy in an externally valid sample size.

In this matched cohort, there were no consistent differences in 90‐day systemic complications between patients with and without diabetes, and overall rates of serious medical events remained low. Only hospital admission differed between groups. Rates of emergency department visits, deep vein thrombosis, anemia, and other measured systemic complications were similar. These findings align with the established safety profile of hip arthroscopy, in which most complications are minor and major events such as deep infection, thromboembolic events, femoral neck fracture, and dislocation are rare.^11^, ^12^ The current body of literature regarding orthopaedic surgery in the setting of diabetes confirms that glycemic control is a critical determinant of postoperative outcomes, with higher rates of postoperative medical complications, impaired wound healing, and readmission linked to hyperglycemia.^3^, ^13^, ^14^ The mean hemoglobin A1c was 6.59 ± 1.64, suggesting generally adequate glycemic control among those with recorded laboratory values. This may have contributed to the low rates of analyzable 90‐day systemic outcomes observed in the present study.^9^, ^15^, ^16^ However, the study was not designed to stratify outcomes by glycemic control. In addition, these findings inform evidence presented by Nicolay et al., who revealed that the combination of diabetes and obesity was predictive of 30‐day postoperative complications in hip, knee, and shoulder arthroscopy, but diabetes alone was not predictive.^8^ The present analysis controlled for the presence of obesity and failed to find a significant impact of diabetes on acute systemic outcomes, indicating that the interaction between obesity and diabetes may be more important than the presence of either comorbidity alone. The presence of diabetes in conjunction with obesity, if accompanied by hypercholesterolemia or hypertension, is named metabolic syndrome. Metabolic syndrome has been well established as a risk for acute systemic complications in total joint arthroplasty,^17^, ^18^ and some evidence implicates metabolic syndrome as a risk factor for higher rates of medical complications after shoulder arthroscopy.^19^ Taken together with the results of the present study, well‐controlled diabetes alone may not be an independent risk factor for complications after hip arthroscopy, and future research may investigate the role of metabolic syndrome and metabolic derangements that impact systemic illness postoperatively.

In contrast to the largely similar systemic profile, several hip‐specific outcomes differed between groups. There were higher rates of subsequent hip arthroscopy, arthroscopic irrigation and debridement, and infection among patients with diabetes, whereas rates of subsequent labral repair were similar. These should be interpreted cautiously. Events occurring at 1 and 3 years may reflect progression of underlying hip pathology, contralateral hip disease, differences in health care use, infection diagnoses unrelated to the index procedure, or true ipsilateral reintervention. Because laterality and clinical documentation are unavailable in TriNetX, these possibilities cannot be distinguished. These findings should also be considered alongside prior studies. Perets et al. reported that diabetic patients achieved similar improvements in patient‐reported outcomes and had comparable revision and arthroplasty conversion rates to matched controls at 2 years.^7^ In contrast, Quesada‐Jimenez et al. evaluated 29 diabetic hips with 75 matched nondiabetic hips at 2 years and found an increased rate of subsequent arthroscopy in the diabetic cohort, with 7 hips (24.1%) requiring subsequent arthroscopy compared with 8 (9.2%) in the control cohort.^10^ Our results coincide with those of Quesada‐Jimenez et al., with diabetic patients more likely to undergo debridement and revision femoroplasty/acetabuloplasty. Regarding infection, our results are similar to those of other database studies showing that diabetes, especially in the presence of obesity or poor glycemic control, increases infection risk after knee and shoulder arthroscopic procedures.^2^, ^20^ Events occurring at 1 and 3 years may reflect progression of underlying hip pathology, contralateral hip disease, differences in health care use, infection diagnoses unrelated to the index procedure, or true ipsilateral reintervention. Because laterality and clinical documentation are unavailable in TriNetX, these possibilities cannot be distinguished. Together, these findings indicate that diabetes may be associated with a higher burden of reintervention and infection at the population level, particularly over longer follow‐up.

Overall, the current analysis has important clinical implications. Serious systemic complications at 90 days remained uncommon and largely similar between groups, supporting the safety of hip arthroscopy in patients with diabetes. However, higher rates of several hip‐specific adverse outcomes, including subsequent arthroscopy, arthroscopic debridement, and infection, were observed over midterm follow‐up. These findings may aid in preoperative counseling and expectation setting, guide perioperative planning, and reinforce the importance of optimizing glycemic control in accordance with contemporary guidelines.^9^, ^15^ Further research linking glycemic metrics, pathology severity, and long‐term patient‐reported outcomes will help refine risk stratification and management strategies for diabetic patients undergoing hip arthroscopy.

### Limitations

Several limitations should be considered. The principal limitation of this analysis is the lack of laterality in the TriNetX platform. Therefore, subsequent hip arthroscopy, labral repair, femoroplasty, acetabuloplasty, and debridement codes cannot be confirmed to represent revision procedures on the index hip and may include contralateral hip procedures. For this reason, the results are framed as subsequent hip‐related procedures after index hip arthroscopy rather than confirmed ipsilateral revisions. This limitation precludes causal inference regarding whether later procedures were directly attributable to the index operation. The use of retrospective administrative data introduces the potential for misclassification of exposures and outcomes. Hemoglobin A1c values within 3 months of arthroscopy were available for only 872 patients, representing 48.9% of the matched diabetes cohort. Consequently, the reported mean hemoglobin A1c may not represent glycemic control across the entire cohort, and selection bias may have been introduced if patients who underwent perioperative A1c testing differed systematically from those without recorded values. The incomplete laboratory data also precluded meaningful stratification of outcomes according to glycemic control. Preoperative and operative findings, such as structural pathology, radiographic parameters, and surgical technique, are unavailable. Patient‐reported outcomes cannot be assessed but were not the focus of this investigation, which targeted objective adverse events. Despite matching on 35 characteristics, unmeasured confounders such as socioeconomic factors, surgeon experience, and rehabilitation adherence may persist.^2^ TriNetX suppresses event counts below 10, limiting assessment of rare but clinically important complications, and our study was likely underpowered to detect these rare complications. Follow‐up is constrained to encounters within participating health systems, which may underestimate procedures performed elsewhere. Finally, sex‐stratified outcome analyses were considered; however, because several outcomes were rare and TriNetX suppresses event counts when 10 or fewer patients experience an outcome, disaggregation by sex would have resulted in substantial censoring of clinically relevant endpoints.

## CONCLUSIONS

Diabetes was not associated with higher rates of most early systemic complications after hip arthroscopy, although hospital admissions were more common among patients with diabetes. Diabetes was associated with higher rates of subsequent hip arthroscopy procedures, arthroscopic irrigation and debridement, and infection diagnoses after index hip arthroscopy; however, these findings should be interpreted in the context of database limitations, including the inability to confirm laterality or ipsilateral revision status.

## SUPPORTING INFORMATION

Additional supporting information can be found online in the Supporting Information section.

## DECLARATION OF GENERATIVE AI AND AI‐ASSISTED TECHNOLOGIES IN THE WRITING PROCESS

During the preparation of this work, the authors used OpenAI ChatGPT to assist with language polishing only. After using this tool, the authors reviewed and edited the content as needed and take full responsibility for the content of the publication.

## DISCLOSURES

The author (B.G.) declares the following financial interests/personal relationships which may be considered as potential competing interests: B.G. reports royalties or licenses; consulting fees (paid consultant); and payment or honoraria for lectures, presentations, speakers bureaus, manuscript writing, or educational events (research support) from Arthrex. The other authors (N.J.M., J.H., J.W.) declare that they have no known competing financial interests or personal relationships that could have appeared to influence the work reported in this paper.

## Supporting information

Supplementary Material
