# Supplementary material for: Diabetes Mellitus Is Associated With Increased Risk for Subsequent Hip Arthroscopy After Index Hip Arthroscopy
Source: Arthrosc Sports Med Rehabil. 2026 Oct 16:e70105. Online ahead of print. doi: 10.1002/ars2.70105 (PMC13674581; doi:10.1002/ars2.70105)
Supplement: Supplementary file 1 [file ARS2-9999-e70105-s001.pdf]

Supporting Information

Supplementary Table 1: Propensity Score Matching

Variable (ICD Code)	*+Diabetes Before Matching N (%)*	*-Diabetes Before N (%)*	*Std Diff Before*	*p Before*	*Hip scope diabetes Before N (%)*	*Hip scope no diabetes Before N (%)*	*Std Diff After*	*p After*
Age at Index	1,831 (100%)	33,550 (100%)	0.846	<0.001	1,784 (100%)	1,784 (100%)	0.064	0.056
Male	653 (35.7%)	11,605 (34.6%)	0.022	0.347	641 (35.9%)	628 (35.2%)	0.015	0.649
White	1,422 (77.7%)	27,991 (83.4%)	0.146	<0.001	1,392 (78.0%)	1,398 (78.4%)	0.008	0.808
Black or African American	204 (11.1%)	1,665 (5.0%)	0.229	<0.001	193 (10.8%)	187 (10.5%)	0.011	0.745
Hispanic or Latino	145 (7.9%)	1,814 (5.4%)	0.101	<0.001	134 (7.5%)	135 (7.6%)	0.002	0.949
Malignant neoplasms (C76–C80)	23 (1.3%)	82 (0.2%)	0.117	<0.001	22 (1.2%)	16 (0.9%)	0.033	0.328
Lymphoid malignancies (C81–C96)	13 (0.7%)	63 (0.2%)	0.078	<0.001	11 (0.6%)	13 (0.7%)	0.014	0.682
Iron deficiency anemia (D50)	120 (6.6%)	596 (1.8%)	0.241	<0.001	105 (5.9%)	87 (4.9%)	0.045	0.182
Coagulation disorders (D65–D69)	70 (3.8%)	480 (1.4%)	0.150	<0.001	65 (3.6%)	63 (3.5%)	0.006	0.857
Other hypothyroidism (E03)	245 (13.4%)	1,158 (3.5%)	0.363	<0.001	224 (12.6%)	215 (12.1%)	0.015	0.646
Overweight and obesity (E66)	555 (30.3%)	2,054 (6.1%)	0.660	<0.001	513 (28.8%)	492 (27.6%)	0.026	0.434
Electrolyte disorders (E87)	201 (11.0%)	568 (1.7%)	0.388	<0.001	177 (9.9%)	164 (9.2%)	0.025	0.459
Mental disorders due to physiologic condition (F06)	17 (0.9%)	79 (0.2%)	0.091	<0.001	15 (0.8%)	15 (0.8%)	<0.001	1.000
Psychoactive substance disorders (F19)	31 (1.7%)	174 (0.5%)	0.112	<0.001	28 (1.6%)	28 (1.6%)	<0.001	1.000
Essential hypertension (I10)	652 (35.6%)	1,783 (5.3%)	0.810	<0.001	607 (34.0%)	610 (34.2%)	0.004	0.916
Atrial fibrillation (I48)	32 (1.7%)	77 (0.2%)	0.154	<0.001	25 (1.4%)	28 (1.6%)	0.014	0.678
Heart failure (I50)	42 (2.3%)	45 (0.1%)	0.198	<0.001	30 (1.7%)	28 (1.6%)	0.009	0.791
Peripheral vascular disease (I73.9)	22 (1.2%)	57 (0.2%)	0.125	<0.001	16 (0.9%)	10 (0.6%)	0.040	0.238

Chronic obstructive pulmonary disease (J44)	57 (3.1%)	126 (0.4%)	0.210	<0.001	43 (2.4%)	27 (1.5%)	0.065	0.053
Rheumatoid arthritis (M06)	58 (3.2%)	324 (1.0%)	0.155	<0.001	51 (2.9%)	54 (3.0%)	0.010	0.766
Chronic kidney disease (N18)	77 (4.2%)	143 (0.4%)	0.253	<0.001	59 (3.3%)	47 (2.6%)	0.040	0.237
Chronic pain syndrome (G89.4)	120 (6.6%)	543 (1.6%)	0.251	<0.001	105 (5.9%)	94 (5.3%)	0.027	0.422
Anxiety disorder (F41.9)	472 (25.8%)	3,988 (11.9%)	0.361	<0.001	444 (24.9%)	442 (24.8%)	0.003	0.938
Generalized anxiety disorder (F41.1)	172 (9.4%)	1,436 (4.3%)	0.204	<0.001	163 (9.1%)	159 (8.9%)	0.008	0.815
Major depressive disorder (F33)	160 (8.7%)	906 (2.7%)	0.262	<0.001	147 (8.2%)	134 (7.5%)	0.027	0.419
Bipolar disorder (F31)	88 (4.8%)	440 (1.3%)	0.204	<0.001	77 (4.3%)	64 (3.6%)	0.037	0.264
Persistent mood disorders (F34)	55 (3.0%)	355 (1.1%)	0.138	<0.001	50 (2.8%)	42 (2.4%)	0.028	0.398
Nicotine dependence (F17)	302 (16.5%)	1,651 (4.9%)	0.381	<0.001	277 (15.5%)	283 (15.9%)	0.009	0.782
Opioid dependence (F11.2)	29 (1.6%)	121 (0.4%)	0.125	<0.001	24 (1.3%)	25 (1.4%)	0.005	0.886
Alcohol related disorders (F10)	58 (3.2%)	377 (1.1%)	0.141	<0.001	57 (3.2%)	61 (3.4%)	0.013	0.708
Cannabis related disorders (F12)	45 (2.5%)	376 (1.1%)	0.101	<0.001	43 (2.4%)	43 (2.4%)	<0.001	1.000
Hypertensive diseases (I10–I1A)	662 (36.2%)	1,808 (5.4%)	0.820	<0.001	616 (34.5%)	619 (34.7%)	0.004	0.916
Cardiomyopathy (I42)	28 (1.5%)	58 (0.2%)	0.148	<0.001	20 (1.1%)	19 (1.1%)	0.005	0.872
Asthma (J45)	339 (18.5%)	3,010 (9.0%)	0.280	<0.001	310 (17.4%)	320 (17.9%)	0.015	0.661
Congenital deformities of hip (Q65)	123 (6.7%)	3,391 (10.1%)	0.122	<0.001	123 (6.9%)	111 (6.2%)	0.027	0.417

Supplementary Table 1 Legend: Table displays variables included in the propensity score matching model m. Columns show the number and percentage of patients with each diagnosis before matching and after matching for each cohort. Standardized differences reflect balance between cohorts, with values < 0.1 indicating good covariate balance. P-values < 0.01 are bolded.
